# Double-Expressor Phenotype (BCL-2/c-MYC Co-expression) of Diffuse Large B-Cell Lymphoma and Its Clinicopathological Correlation

**DOI:** 10.7759/cureus.13155

**Published:** 2021-02-05

**Authors:** Atif A Hashmi, Syeda N Iftikhar, Gul Nargus, Omer Ahmed, Ishaq Azeem Asghar, Umme Aiman Shirazi, Anoshia Afzal, Muhammad Irfan, Javaria Ali

**Affiliations:** 1 Pathology, Liaquat National Hospital and Medical College, Karachi, PAK; 2 Pathology, Khyber Medical College, Peshawar, PAK; 3 Internal Medicine, Liaquat National Hospital and Medical College, Karachi, PAK; 4 Pathology, Ascension St. John Hospital, Detroit, USA; 5 Pathology, University of Oklahoma Health Sciences Center, Oklahoma City, USA; 6 Statistics, Liaquat National Hospital and Medical College, Karachi, PAK

**Keywords:** diffuse large b-cell lymphoma, germinal center subtype, non-germinal center subtype, double-expressor, c-myc, bcl-2

## Abstract

Introduction

Diffuse large B-cell lymphoma (DLBCL) is a heterogeneous disease, the spectrum of which is increasing with time. The 2016 World Health Organization (WHO) update on hematopoietic tumors recognized a prognostic subgroup of DLBCL called double-expressor DLBCL. Double-expressor DLBCL is defined by the co-expression of c-MYC and BCL-2 by using immunohistochemical (IHC) studies. To our knowledge, very few studies have looked into the pathological features of this newly defined prognostic category of DLBCL; therefore, in this study we evaluated the frequency of the double-expressor phenotype of DLBCL and its association with other clinicopathological parameters.

Methods

We conducted a retrospective observational study in the Department of Histopathology, Liaquat National Hospital and Medical College, from November 2017 till December 2020. Pathological and clinical records were retrieved from departmental archives. All cases diagnosed as DLBCL were included in the study. More than 40% c-MYC expression in the presence of more than 50% BCL-2 expression was defined as double-expressor DLBCL.

Results

The mean age of the patients was 52.1±16.9 years. The mean Ki67 index was 73.0±17.0%. A total of 48.6% cases were of germinal center B-cell-like (GCB) subtype, and 59.6% cases were nodal. Double-expressor phenotype was noted in 35.8% of DLBCL cases. A significant association of double-expressor phenotype was noted with age, gender, Ki67 index and subtype of DLBCL. Double-expressor DLBCL had a higher mean age than non-double-expressor DLBCL. Similarly, double-expressor DLBCL had a higher Ki67 index. Moreover, double-expressor phenotype was associated with non-GCB subtype DLBCL.

Conclusion

We found a high proportion of double-expressor phenotype DLBCL in our population. Moreover, double-expressor phenotype DLBCL was associated with female gender, higher age, higher Ki67 and non-GCB subtype. The association of double-expressor DLBCL with a high Ki67 index and non-GCB subtype confers a poor prognostic significance of this variant of DLBCL, requiring more aggressive therapy.

## Introduction

Diffuse large B-cell lymphoma (DLBCL) is a heterogeneous disease, the spectrum of which is increasing with time [1]. Gene expression profiling studies have distinguished two subtypes of DLBCL, namely, germinal center B-cell-like (GCB) and activated B-cell-like (ABC) subtypes. While molecular studies are considered the gold standard for this subtyping, immunohistochemical (IHC) studies are considered surrogate for the subtyping of DLBCL into GCB and non-GCB types. Studies have shown the prognostic significance of this subtyping, with the GCB subtype being prognostically better than the non-GCB subtype [2]. The 2016 World Health Organization (WHO) update on hematopoietic tumors recognized another prognostic subgroup of DLBCL called double-expressor DLBCL. Double-expressor DLBCL is defined by the co-expression of c-MYC and BCL-2 by using IHC studies. Double-expressor DLBCL is prognostically poorer than the non-double expressor phenotype DLBCL; however, it is better than double-hit and triple-hit B-cell lymphomas, prognostically. Double-hit and triple-hit B-cell lymphomas are high-grade B-cell lymphomas defined by the genetic rearrangements of c-MYC and BCL-2 and/or BCL-6, accordingly, by targeted fluorescence in situ hybridization (FISH) studies. Double-hit and triple-hit lymphomas are currently not considered subtypes of DLBCL as they prognostically behave differently than DLBCL. DLBCL is the most common non-Hodgkin’s lymphoma in Pakistan [3]. To our knowledge, very few studies have looked into the pathological features of this newly defined prognostic category of DLBCL; therefore, in this study we evaluated the frequency of the double-expressor phenotype of DLBCL and its association with other clinicopathological parameters.

## Materials and methods

**Figure 1:**
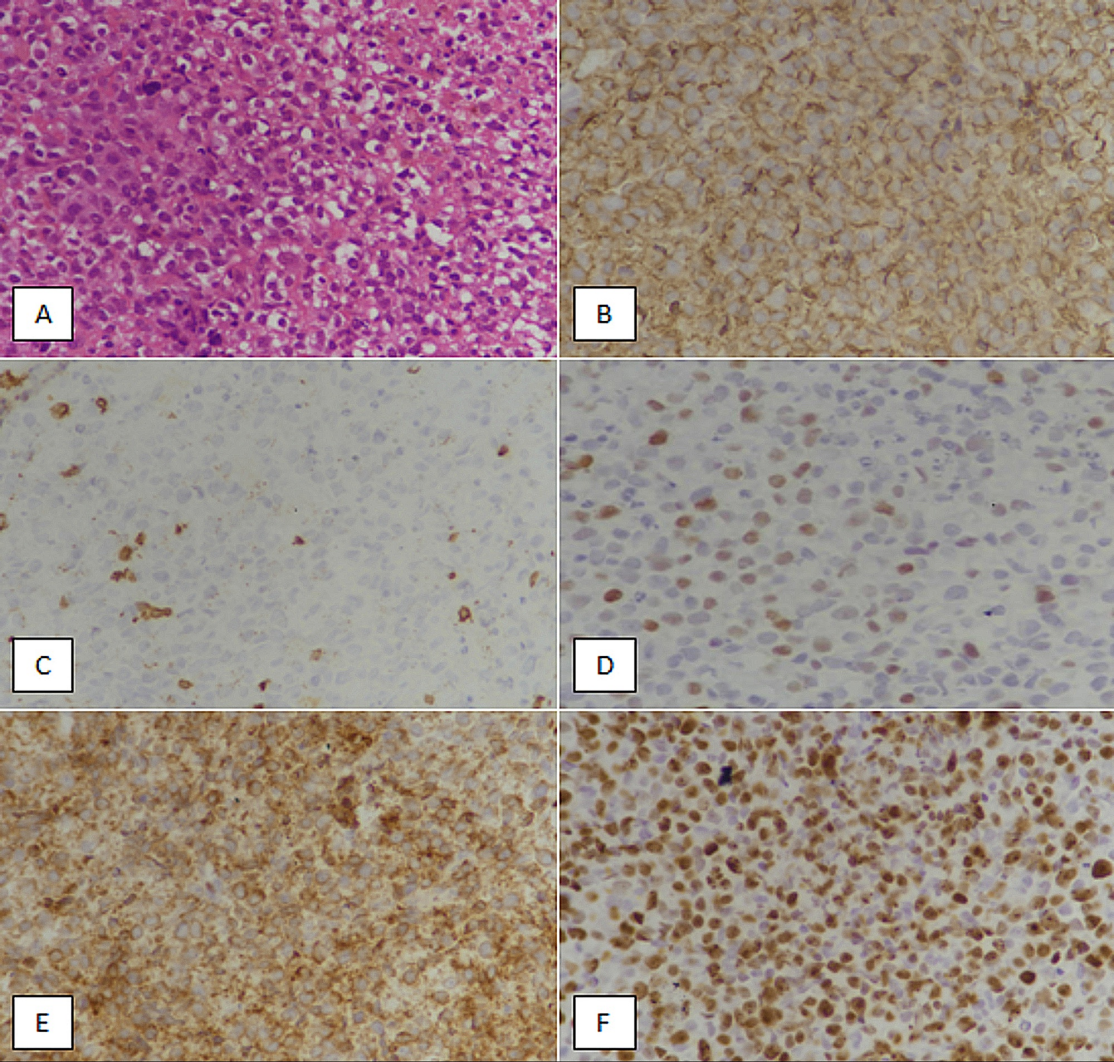
Double-expressor diffuse large B-cell lymphoma (A) Hematoxylin and eosin-stained section at 400x magnification shows sheets of large atypical cells. (B) CD20 immunohistochemical staining shows diffuse membranous expression in atypical lymphoid cells. (C) CD3 immunostaining reveals no expression in tumor cells. Occasional background reactive T-cells are highlighted. (D) c-MYC immunohistochemical staining is depicting positive nuclear expression in more than 40% of the tumor cells. (E) BCL-2 immunohistochemical staining shows diffuse expression in lymphoma cells. (F) The Ki67 immunomarker is revealing an 85% proliferative index.

We conducted a retrospective observational study in the Department of Histopathology, Liaquat National Hospital and Medical College, from November 2017 till December 2020. Pathological and clinical records were retrieved from departmental archives. All cases diagnosed as DLBCL were included in the study. A panel of IHC stains was applied to diagnose DLBCL, including CD20, PAX5, CD3, CD5, SOX11, cyclin D1, CD10, and CD23. If differential diagnosis included carcinoma or melanoma, then S100 and pan-cytokeratin IHC stains were included in the initial IHC panel. Further IHC studies were performed to subcategorize DLBCL. The Hans algorithm was used to subcategorize DLBCL into GCB and non-GCB subtypes. GCB subtype was defined as more than 30% expression of CD10, or more than 30% expression of BCL-6 in the absence of MUM1 and CD10 expression. All other immunophenotypic expressions (CD10-/MUM1+/BCL-6+, CD10-/MUM1+/BCL-6-, CD10-/MUM1-/BCL-6-) were labeled as non-GCB DLBCL. More than 40% c-MYC expression in the presence of more than 50% BCL-2 expression was defined as double-expressor DLBCL (Figure 1).

Data analysis was performed using the Statistical Package for the Social Sciences, version 26.0 (IBM Corp., Armonk, NY). Chi-square test, independent t-test, and Fisher’s exact test were used to check the association. p-values < 0.05 were considered as significant.

## Results

A total of 109 DLBCL cases were included in the study. The mean age of the patients was 52.1±16.9 years. The mean Ki67 index was 73.0±17.0%. A total of 48.6% cases were of GCB subtype, and 59.6% cases were nodal. Double-expressor phenotype was noted in 35.8% of DLBCL cases (Table 1).

**Table 1:** Descriptive statistics of the study population DLBCL, diffuse large B-cell lymphoma *Mean±standard deviation

Clinicopathological characteristics	Frequency (%)
Age (years)*	52.1±16.9
Ki67 (%)*	73.0±17.0
Age groups	
≤35 years	21 (19.3)
36-50 years	23 (21.1)
>50 years	65 (59.6)
Gender	
Male	59 (54.1)
Female	50 (45.9)
Subtype of DLBCL	
Germinal center B-cell-like subtype	53 (48.6)
Non-germinal center B-cell-like subtype	56 (51.4)
Site	
Nodal	65 (59.6)
Extra-nodal	44 (40.4)
Specimen type	
Trucut biopsy	49 (45)
Excision biopsy	60 (55)
BCL-2	
Positive	66 (60.6)
Negative	43 (39.4)
BCL-6	
Positive	51 (46.8)
Negative	58 (53.2)
MUM1	
Positive	46 (42.2)
Negative	63 (57.8)
c-MYC	
Positive	48 (44)
Negative	61 (56)
CD10	
Positive	44 (40.4)
Negative	65 (59.6)
CD30	
Positive	9 (8.3)
Negative	100 (91.7)
Double-expressor phenotype	
Yes	39 (35.8)
No	70 (64.2)

A significant association of double-expressor phenotype was noted with age, gender, Ki67 index, and subtype of DLBCL. Double-expressor DLBCL had a higher mean age than non-double-expressor DLBCL. Similarly, double-expressor DLBCL had a higher Ki67 index. Moreover, double-expressor phenotype was associated with non-GCB-type DLBCL. No significant association was noted with respect to the site of involvement (nodal vs. extra-nodal) and CD30 IHC expression (Table 2).

**Table 2:** Association of double-expressor phenotype DLBCL with clinicopathological features DLBCL, diffuse large B-cell lymphoma *Mean± standard deviation; an independent t-test was applied. **A chi-square test was applied. ***Fisher’s exact test was applied. ****p-value significant at <0.05.

Clinicopathological characteristics	Frequency (%)		p-value
	Double-expressor phenotype DLBCL		
	Yes	No	
Age (years)*	59.4±12.6	48.0±17.8	<0.0001****
Ki67 (%)*	78.1±15.2	70.2±17.4	0.019****
Age groups**			
≤35 years	3 (7.7)	18 (25.7)	<0.0001****
36-50 years	3 (7.7)	20 (28.6)	
>50 years	33 (84.6)	32 (45.7)	
Gender**			
Male	13 (33.3)	46 (65.7)	0.001****
Female	26 (66.7)	24 (34.3)	
Subtype of DLBCL**			
Germinal center B-cell-like subtype	14 (35.9)	39 (55.7)	0.047****
Non-germinal center B-cell-like subtype	25 (64.1)	31 (44.3)	
Site**			
Nodal	25 (64.1)	40 (57.1)	0.478
Extra-nodal	14 (35.9)	30 (42.9)	
BCL-6**			
Positive	21 (53.8)	30 (42.9)	0.270
Negative	18 (46.2)	40 (57.1)	
MUM1**			
Positive	26 (66.7)	20 (28.6)	<0.0001****
Negative	13 (33.3)	50 (71.4)	
CD10**			
Positive	10 (25.6)	34 (48.6)	0.019****
Negative	29 (74.4)	36 (51.4)	
CD30***			
Positive	3 (7.7)	6 (8.6)	1.000
Negative	36 (92.3)	64 (91.4)	

## Discussion

In this study, we found that a significant proportion of DLBCL had co-expression of BCL-2 and c-MYC conferring a double-expressor phenotype according to the WHO classification. Moreover, double-expressor phenotype DLBCL was associated with female gender, higher age, higher Ki67 index and non-GCB subtype.

Some studies have proposed that the prognosis of c-MYC/BCL-2 double-expressor phenotype is worse than other subtypes of DLBCL [4,5], but other studies had mixed results [6,7]. Nagib et al. studied double-expressor DLBCL and found that it was associated with an overall decreased disease-free survival [8]. Naseem et al. studied the frequency and prognosis of double-expressor DLBCL and found that the frequency of c-MYC/BCL-2 co-expression was 14%, with a median survival of 10 months [9]. Conversely, we found a higher frequency of double-expressor phenotype in our study (35.8%).

It is recommended to differentiate double-expressor DLBCL from other DLBCL subtypes as they have poor clinical behavior and need more aggressive interventions [10,11]. Some recent studies have confirmed that a high Ki67 proliferation index and c-MYC/BCL-2 co-expression in DLBCL were independently associated with poor clinical outcomes [2,12,13]. Factors such as age, sex, and proliferation index and their relationship with DLBCL subtypes should be studied in detail to fully understand the importance of each factor separately, as it might have significant clinical implications.

Owing to the expression of cMYC, the differential diagnosis of double-expressor DLBCL also includes Burkitt’s lymphoma (BL). However, BL is characterized by intermediate-sized lymphoid cells with almost 100% Ki67 index, positive expression with CD10 and BCL-6, and lack of expression of BCL-2, although the same immuophenotype can be encountered in DLBCL and differentiation is sometimes difficult to make in the absence of molecular/genetic analysis.

Standard therapy for DLBCL is rituximab, cyclophosphamide, hydroxydaunorubicin (doxorubicin), Oncovin (vincristine), and prednisone (R-CHOP). Owing to the aggressive nature of double-expressor and double-hit lymphoma, more aggressive regimens than R-CHOP like EPOCH (etoposide, vincristine, doxorubicin, with cyclophosphamide and prednisone) were suggested; however, the prognosis remains dismal. Alternatively, novel-targeted agents, directly or indirectly inhibiting BCL-2 and cMYC, are being investigated and results are encouraging [14].

Our study was limited owing to the small sample size and retrospective study design. Moreover, clinical follow-up data were not available to compare survival between double-expressor and non-double expressor phenotypes of DLBCL in our study. In addition, molecular studies were not performed to evaluate genetic rearrangements of c-MYC and BCL-2.

## Conclusions

In this study, we evaluated the co-expression of BCL-2 and c-MYC in DLBCL designated as the double-expressor phenotype DLBCL. We noted that a significant proportion of DLBCL in our study had double-expressor phenotype. We also found that double-expressor DLBCL had a higher Ki67 index than the non-double-expressor DLBCL. An association of double-expressor phenotype was also noted with non-GCB-type DLBCL. As previous studies have confirmed a poor prognostic significance of a high Ki67 index and non-GCB type in DLBCL, an association of double-expressor DLBCL with non-GCB phenotype and a high Ki67 index portends a poor prognostic significance of double-expressor DLBCL. Future studies are warranted to evaluate the difference in disease-free survival of double-expressor DLBCL and other subtypes of DLBCL.
